# Moderate Resistive Training Maintains Bone Mineral Density and Improves Functional Fitness in Postmenopausal Women

**DOI:** 10.4061/2010/760818

**Published:** 2010-06-13

**Authors:** Danilo Sales Bocalini, Andrey Jorge Serra, Leonardo dos Santos

**Affiliations:** ^1^Unifesp-EPM, Rua Coronel Lisboa, 819, 04020-041 São Paulo, Brazil; ^2^Incor, Rua Dr Eneis Carvalho Aguiar, 44, 10 andar, 05403-000 São Paulo, Brazil

## Abstract

Twenty five subjects were randomized to untrained (UN) and resistive-trained (RT) groups. The RT group exercised three sessions per week at 60%–70% of the load according to individual 1RM test during 24 weeks. Both groups were evaluated before and after protocol period assessing lumbar spine (LS) and femoral neck (FN) BMD by dual-energy X-ray absorptiometry, VO_2_ max, and neuromuscular fitness. After 24 weeks, there were significant reductions in LS (0.89 ± 0.16% loss) and FN BMD (1.54 ± 0.35% loss) for UN but no change was found in the TR (LS: 0.01 ± 0.12% and FN: 0.04 ± 0.05% loss). The UN group had no changes in neuromuscular performance. However, RT exhibited a significant improvement on the functional fitness parameters evaluated, with the exception of agility. Our results indicate RT suppresses the decline in BMD and simultaneously improves the functional fitness of postmenopausal women without hormone replacement therapy, which may reduce fall risk and related bone fractures.

## 1. Introduction

Aging is often associated with reductions in bone mineral density (BMD) and muscle mass [1] and, consequently, with higher risk for falls and bone fractures [2].Currently, bone demineralization (osteopenia) and muscle loss (sarcopenia) affect over 34 million people which results in more than 2 million bone fractures a year in the United States (NIH/NIAMS, 2009). With aging, while both sexes lose bone, women exhibit loss rates substantially higher than men, suffering a greater risk for osteoporosis after menopause [3–5]. Some studies suggest that the reduced ovarian function and serum hormones levels accelerate the rate of bone loss and may also be involved in the decline of muscle mass and strength [6].

Basic exercise guidelines recommended by the American College of Sports Medicine for healthy adults and elderly people emphasize that training programs consist of resistance, strength, aerobic, and flexibility exercises. Resistive training (RT) is an exercise modality that imposes heavy loads upon the skeleton and, consequently, should increase both body strength and muscle mass in older women. Similar to other studies [1, 7, 8] our group demonstrated that intense RT promotes several benefits in postmenopausal women such as improved body composition parameters and muscular strength with a significant preservation of BMD [9]. However, the literature remains equivocal, since other investigators did not report such findings [10–12]. Furthermore, the effectiveness of resistive training on the bone health simultaneously evaluated with functional fitness tests in postmenopausal women who are not using drugs that affect bone or muscle metabolism, is unknown.

Thus the aim of this study was to evaluate the influences of moderate resistive training on the BMD, as well as the efficacy to improve functional fitness parameters in postmenopausal women without hormone replacement therapy. 

## 2. Material and Methods

### 2.1. Participants

Thirty volunteer women (age of 57 to 75 years) were recruited from the Regional Community Adult Day Care facilities located around the UNIFESP Hospital. All participants had medical examinations and completed questionnaires regarding medical history, and protocols were performed within accordance with the ethical standards of the Helsinki Declaration (1964, amended in 1975 and 1983). Inclusion criteria were age of more than 55 years old, completed menopause for at least two years before, and no use of hormone replacement therapy, or drugs and vitamins that could alter calcium or bone metabolism. Exclusion criteria were as follows: participation in current or previous regular exercise training in the last 6 months; recent hospitalization; symptomatic cardiorespiratory disease; hypertension or metabolic syndrome; severe renal or hepatic disease; cognitive impairment or progressive and debilitating conditions; marked obesity with inability to exercise; recent bone fractures or any other medical contra-indications to training. According to the exclusion criteria, 5 women could not participate. Consequently, 25 women were randomized for untrained (UN; *n* = 12) or resistive trained (RT; *n* = 13) group. For the RT group, participation in less than 90% of the stipulated exercise program was also considered as exclusion criteria. The UN group continued only with their usual daily activities. All of the subjects were instructed to maintain their previously controlled normal dietary intake, to inform any new prescribed medication, and finally to not participate in any other type of physical activity.

### 2.2. Evaluated Parameters

#### 2.2.1. Body Composition and Anthropometry

Height (m) and weight (kg) were measured to calculate body mass index (BMI = weight/height^2^). Body fat percentage was derived with skin folds as previously proposed [13].

#### 2.2.2. Bone Mineral Density

Dual-energy X-ray absorptiometry (DEXA) was used to scan the lumbar spine (L_1_–L_4_) and the proximal femoral neck. All data were analyzed by same investigator using DEA – DTX 200 (Osteometer MediTech), blind to the subject group.

#### 2.2.3. Maximum Aerobic Power

The Bruce treadmill protocol (increments in both speed and grade in 3-minute intervals) was chosen to determine VO_2_ max. A one-minute warm-up at 1.0 mph without treadmill inclination preceded the Bruce treadmill protocol. Velocity and inclination were adjusted gradually as previously described [14]. The subjective level of exertion was assessed using the Borg 6-to-20 scale, and the exercise test was stopped due to exhaustion or according to previously described criteria [14]. Test was considered satisfactory if the participants reached the age-graded maximal recommended heart rate (220—age in years) without stoppage criteria. The VO_2_ max was calculated as proposed by the American College of Sports Medicine [14].

#### 2.2.4. Muscle Strength

The muscular strength was evaluated by the chest press and leg extension. Upper and lower body muscle strength were assessed by 1 RM. Successive attempts were made with 1 to 2 minutes rest between attempts until failure as previously described [9].

#### 2.2.5. Functional Fitness

 This evaluation was composed by six tests previously reported in the literature to assess physical performance parameters concerning mobility and balance in older adults [15–18]. The arm curl test was used to evaluate upper limb fitness, with the analyzed score as the total number of hand weight curls through the full range of motion; the chair stand test was used to evaluate lower limbs strength, scored by the number of standing up executed correctly within 30 seconds. Agility was evaluated by the 8-foot up-and-go test, and the score was considered as the shortest time to rise from a seated position, walk eight feet, turn back and return to the seated position. The sit and reach test was used to evaluate the lower body flexibility scored by the shortest distance achieved between the extended fingers and the toe when seated with extended leg and the heel resting on the floor. The back scratch test assessed the upper body flexibility and the score was considered the shortest distance achieved between the extended middle fingers when reaching behind the head with one hand and behind the back with the other hand. Static balance was assessed by having subjects stood up on just one leg for a maximum of 30 seconds on each side. The score was taken considering the time of quiet standing up, allowing only minimal fluctuations of ankle position or obvious toe clawing, without hopping or upper limbs movement. The test was stopped after 30 seconds if hopping occurred, the ankle movement was excessive, or the hanged foot touched the floor or contacted the stance leg/foot.

### 2.3. Moderate Resistive Training Program

The resistive training program consisted of one-hour exercise sessions 3 times a week in nonconsecutive days for 24 weeks [9]. Each session included the following isotonic exercises: leg press, leg extension, leg curl, chest press, elbow flexion, elbow extension, upper back row, and abdominal flexion. On the first week all subjects from RT group started the exercise program at 40% of 1 RM for each given exercise. The load was gradually increased until the subjects could perform three sets of 10–12 repetitions for the given exercise at 60%–70% of 1 RM, considered as moderate intensity [19]. When necessary; adjustments of loads were made every two weeks to promote muscle strength gains. The participants alternated between upper and lower body exercises to minimize fatigue, with rest between sets for 1 minute but no pauses between repetitions. Each session was guided by trained fitness instructors and supervised by the researches.

### 2.4. Statistical Analysis

Analysis of comparisons between groups along the time periods were performed with 2-way ANOVA with repeated measures, followed by Bonferroni's post-hoc test. Comparisons between groups concerning relative changes in variables after 24-weeks were performed by unpaired Student's *t*-test. Statistical analyses were performed with GraphPad Prism software (version 4.0, San Diego, CA, USA). Statistical significance was established at *P* < .05.

## 3. Results

There were no injuries as a result of this workout. For all the evaluated parameters, baseline values were similar between UN and RT group with satisfactory subject homogeneity. 

Table 1 summarizes anthropometric and body composition parameters. After 24 weeks, there were no significant changes in body weight, BMI, and body fat percentage of the UN group, while the RT group exhibited body weight reductions and discreet, but not significant, increments of lean mass.

**Figure 1:**
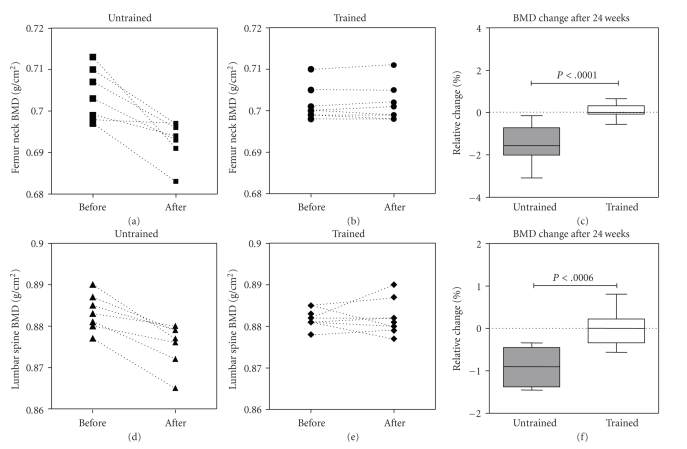
Levels of BMD in femur neck (a and b) and lumbar spine (d and e) before and after the 24-week followup showing reduction in the untrained and no change in the trained group (*P* < .05 for time factor and for interaction between time and group factors by 2-way ANOVA with repeated measures separately for each body site). (c) and (f) The relative changes of BMD in the untrained (filled boxes) and resistive trained women (empty boxes). *P* was calculated by unpaired Student's *t *test.

In the UN group there was a significant reduction in lumbar spine (Figure 1(d), before: 0.883 ± 0.007 versus after: 0.875 ± 0.008 g/cm^2^, *P* < .05) and femoral neck BMD (Figure 1(a), before: 0.704 ± 0.006 versus after 0.693 ± 0.005 g/cm^2^, *P* < .001) after 24 weeks. However, both BMD of lumbar spine (Figure 1(e)) and femoral neck (Figure 1(b)) did not change significantly in RT group (LS before: 0.881 ± 0.002 versus after: 0.882 ± 0.004 g/cm^2^ and FN before: 0.701 ± 0.004 versus after: 0.004 g/cm^2^). As a result, the percentage of BMC decrease in the lumbar spine and femoral neck was significantly different between UN and RT groups (Figures 1(f) and 1(c)), indicating a maintenance of the bone mineral content after resistive training program.

Concerning muscle strength evaluated by the 1 RM test, the trained group demonstrated improvements of 23% in lower limb strength (legs) (before: 36 ± 4 kg versus after: 47 ± 3 kg, *P* < .001) and 32% in upper body strength (before: 21 ± 5 kg versus after: 31 ± 8 kg, *P* < .001). Nevertheless, in the UN group, no changes were observed either on the lower limbs (before: 37 ± 7 kg versus after: 38 ± 4 kg, *P* > .05) or on the arms strength (before: 22 ± 8 kg versus after: 22 ± 9 kg, *P* > .05).

Baseline and 24-week evaluation of maximum aerobic power (VO_2_ max) and functional fitness parameters are presented in Table 2. VO_2_ max remained unchanged after 24 weeks in both groups, and no difference was found between UN and RT. Nevertheless, the resistive training resulted in a substantial improvement of upper limb and lower body muscular strength as evaluated by the arm curl and chair stand tests, respectively. Although agility was unchanged by this exercise program, the RT group exhibited a significant increment on the lower and upper body flexibility, as well as on the static balance. On the other hand, no changes occurred to the UN group for any functional fitness parameters.

## 4. Discussion

The benefits of resistive training on the bone demineralization in postmenopausal women remain uncertain. Therefore, the main contribution of the present study was to reinforce evidence that moderate intensity RT can contribute positively to bone health, muscle strength, and functional fitness of postmenopausal women in the absence of hormone replacement therapy. This maintenance of BMD is clinically very important for postmenopausal women for whom the risk of falls and bone fractures are significantly higher due to advancing age and muscular and bone weakness. Taken together, these conditions often lead elderly people to functional dependence and impairment of quality of life [20].

As mentioned before, studies that evaluated the effects of RT on BMD reported equivocal results. Some authors observed no differences in BMD of lumbar spine [10–12] and femoral neck [11, 12] in trained older women compared to untrained group. On the other hand, several studies demonstrated positive effects of RT on the BMD [1, 7, 8, 20] and similar results were reported in our previous study [9] using high intensity RT (85% by 1 RM). Further than maintenance, it was described a slight increase in BMD of the femoral neck and lumbar spine after one year of high intensity strength training in postmenopausal women life [20].

The exact physiologic mechanism for which resistive training preserves the BMD is not completely understood. Nevertheless, bone strain was suggested as playing a key role as mediator of the relationship between loading forces and bone remodeling [21]. The piezoelectric effect in bone could justify the maintenance increase of mineral content in these cases [22]. By this mechanism, actions like compression, tension, sprain, or shear stresses can generate differences on electric potential and create a magnetic field on the specific sites of the bone, stimulating cellular activity and consequently mineral deposition in the stress points [22].

Other important aspects should be considered for this BMD improvement, such as the magnitude of the stimuli, which has been described as more important than the frequency of the stimuli [23]. Thus, strength training is more effective in increasing or maintaining BMD when compared to running, already known as a good osteogenic enhancer, especially in anatomical sites where both activities produce mechanical stress, such as the femur neck [24]. This difference is often attributed to the relationship between bone metabolism and muscle strength levels [25]. We recently suggested that emphasis in eccentric muscle action influences positively the outcomes of bone mineralization [9]. In fact, this type of muscle training has shown capable to promote larger osteogenic stimulus when compared to concentric muscle actions [26].

To date, no study has evaluated the simultaneous effects of a resistive training on BMD and functional fitness at postmenopausal women. According to our results, the exercise program not only coursed with the preservation of BMD, but also significantly improved the functional fitness of the trained group. Falls are responsible by approximately 90% of bone fractures in older women and undoubtedly represent an important public health issue [27]. In the elderly, falls are not random events and occur, at least in part, due to physiological dysfunction such as impaired balance, muscular weakness, and impaired reaction time [28, 29]. Furthermore, postmenopausal women suffer more fall-related fractures due to greater impairments in balance and muscular strength combined with osteopenia or osteoporosis [30].

Fall prevention in older people, especially in women, is mainly searched through nonpharmacological strategies such as evaluating vision and hearing, promoting safety hazards at home, and finally, inducing exercise practice [5, 30, 31]. In fact, exercise modalities which improve agility, strength, and balance may significantly reduce the risk of falls and subsequent fracture, independent of any increase in bone density [5]. Despite no increment identified on the VO_2_ max of RT group, our results corroborate the standpoint of the American College of Sports Medicine supporting the idea that participation in regular exercise programs is directly related to improvements of muscle strength, flexibility, and range of motion (ACSM, 1998). In addition, it is recommended that exercise should be adapted to maximize muscular strength, and that resistive training is safe and well tolerated in the elderly, eliciting adaptive neuromuscular changes to physiological variables associated with the risk of fall and disability [32].

In the current study, we also observed RT effectively increased both upper and lower limb strength. This data agrees with one study that found strong association between BMD maintenance with strength improvement to anatomically related bone structures [1]. Muscular strength increases are imperative to enhance the ability to carry out daily tasks, such as standing up from a chair or carrying a box [33]. Moreover, there was considerable improvement of the static balance of trained women. Thus, the moderate resistive training led to significant improvement of lower body and limb strength combined with improved static balance, which has been defined as an independent contributor to fall prevention [34]. Interestingly, although were not performed specific activities for joint flexibility, we identified improvement of both the lower and upper body flexibility. This result reinforces the helpfulness of a moderate intensity program, since flexibility lost are associated with development of musculoskeletal impairment and progressive disabilities in old age [17]. Taken together, enhancement of all these functional parameters induced by RT should be useful in reducing the risk of falls [29]. Contrasting with [30], we have found no difference on the scores for agility test after RT in older women. Possible reasons for this divergence are the different intensity and duration of the training program as well as the agility component of exercise training used by those authors.

In the present study, some limitations must be considered. This is a relatively small-sampled and short-term study without long-term follow-up data. Moreover, the identified positive outcomes with averaged age of 66 in women do not allow these findings to be generalized to any aging population of both genders. Finally, there are some pitfalls in estimating the maximal aerobic power (indirect measurement of VO_2_ max) instead of precisely measuring it through the cardiopulmonary gas exchange approach, although these inconveniences certainly involved both the baseline exercise capacity and that changes in exercise capacity after the RT program.

## 5. Conclusion

The present study suggests moderate resistive training is able to restrain BMD declines of lumbar spine and femur neck in postmenopausal women, even in the absence of hormone replacement therapy, by preventing or attenuating the bone loss. Notably, the exercise program also improved several parameters of functional fitness, including strength, flexibility, and static balance. Thus, despite inherent limitations, our data reinforces the safety and utility of this exercise modality for use in larger trials as well as an adjunctive approach for fall and fractures reduction in this population.

## Figures and Tables

**Table 1:** Anthropometric characteristics.

Parameters	Untrained		Trained	
	Before	After	Before	After
Age (year)	64 ± 8	—	66 ± 9	—
Stature (m)	1.56 ± 0.06	—	1.55 ± 0.04	—
Weight (kg)	69.1 ± 2.2	68.6 ± 2.3	67.9 ± 1.3	64.2 ± 1.1^#†^
BMI (kg/m^2^)	29 ± 2.2	28 ± 2.1	28 ± 1.3	27 ± 1.2
Body fat (%)	30 ± 1.3	31 ± 2.1	30 ± 2.4	28 ± 1.1
Lean mass (kg)	48 ± 1.3	46 ± 2.2	46 ± 2.4	48 ± 1.3

Values are presented as mean ± standard deviation of untrained (*n* = 12) and trained groups (*n* = 13). BMI: body mass index. Two-way ANOVA with repeated measures was used, followed by Bonferroni's post-hoc test. ^#^ *P* < .05 before versus after; ^†^ *P* < .05 untrained versus trained groups.

**Table 2:** Maximal aerobic power (VO_2_ max) and functional fitness parameters.

Parameters	Untrained		Trained	
	Before	After	Before	After
VO_2_ max (ml · kg^−1^ · min ^−1^)	20.0 ± 0.3	20.0 ± 0.3	20.1 ± 0.3	20.1 ± 0.3
Arm curl test (rep)	19.2 ± 0.4	19.0 ± 1.0	19.0 ± 1.2	26.0 ± 1.0^#‡^
Chair stand test (rep)	18.5 ± 1.0	19.0 ± 1.0	19.0 ± 1.0	27.0 ± 1.0^#‡^
8-foot up-and-go test (s)	9.0 ± 1.0	9.0 ± 1.0	9.4 ± 0.5	9.0 ± 0.4
Sit and reach test (cm)	23.0 ± 0.4	22.1 ± 1.0	23.8 ± 0.7	27.1 ± 0.5^#‡^
Back scratch test (cm)	−10.0 ± 0.4	−10.1 ± 1.1	−9.1 ± 0.3	−12.1 ± 0.3^#†^
Static balance (s)	9.0 ± 0.4	8.6 ± 0.8	8.1 ± 0.6	15.0 ± 0.3^#‡^

Values are presented as mean ± standard deviation of untrained (*n* = 12) and trained groups (*n* = 13). Two-way ANOVA with repeated measures was used, followed by Bonferroni's post-hoc test. ^#^ *P* < .001 before versus after; ^†^ *P* < .05 and ^‡^ *P* < .001 untrained versus trained group.
